# Absence of the Yeast Hsp31 Chaperones of the DJ-1 Superfamily Perturbs Cytoplasmic Protein Quality Control in Late Growth Phase

**DOI:** 10.1371/journal.pone.0140363

**Published:** 2015-10-14

**Authors:** Ingo Amm, Derrick Norell, Dieter H. Wolf

**Affiliations:** Institut für Biochemie, Universität Stuttgart, Pfaffenwaldring 55, Stuttgart, Germany; Louisiana State University Health Sciences Center, UNITED STATES

## Abstract

The *Saccharomyces cerevisiae* heat shock proteins Hsp31, Hsp32, Hsp33 and Hsp34 belong to the DJ-1/ThiJ/PfpI superfamily which includes the human protein DJ-1 (PARK7) as the most prominent member. Mutations in the DJ-1 gene are directly linked to autosomal recessive, early-onset Parkinson’s disease. DJ-1 acts as an oxidative stress-induced chaperone preventing aggregation and fibrillation of α-synuclein, a critical factor in the development of the disease. *In vivo* assays in *Saccharomyces cerevisiae* using the model substrate ΔssCPY*Leu2myc (ΔssCL*myc) as an aggregation-prone misfolded cytoplasmic protein revealed an influence of the Hsp31 chaperone family on the steady state level of this substrate. In contrast to the ubiquitin ligase of the N-end rule pathway Ubr1, which is known to be prominently involved in the degradation process of misfolded cytoplasmic proteins, the absence of the Hsp31 chaperone family does not impair the degradation of newly synthesized misfolded substrate. Also degradation of substrates with strong affinity to Ubr1 like those containing the type 1 N-degron arginine is not affected by the absence of the Hsp31 chaperone family. Epistasis analysis indicates that one function of the Hsp31 chaperone family resides in a pathway overlapping with the Ubr1-dependent degradation of misfolded cytoplasmic proteins. This pathway gains relevance in late growth phase under conditions of nutrient limitation. Additionally, the Hsp31 chaperones seem to be important for maintaining the cellular Ssa Hsp70 activity which is important for Ubr1-dependent degradation.

## Introduction

Misfolding of proteins is a process which occurs permanently in the cell. Reasons for the appearance of misfolded proteins are for example genetic mutations, transcriptional or translational errors, interference with metabolic by-products or different environmental stress conditions. These include heat, heavy metal ions or reactive oxygen species (ROS). As accumulated misfolded proteins can be detrimental to cells causing severe diseases in humans, all proteins have to be constantly subjected to quality control, a process which finally decides on the fate of corresponding proteins. Chaperones are essential protein species in the cell fulfilling several tasks in this quality control system. First, partially folded or misfolded proteins exposing hydrophobic patches have to be shielded from the aqueous environment, by this preventing aggregation. Chaperones providing ATPase activity assist in refolding or exhibit disaggregase activity to resolubilize protein aggregates [1–6]. Terminally misfolded proteins which cannot be refolded are degraded by the ubiquitin-proteasome system (UPS) [7–9]. In case of misfolded cytoplasmic proteins in *Saccharomyces cerevisiae* the main ubiquitin ligase (E3) involved in ubiquitination of such substrates for subsequent proteasomal degradation is the RING ligase Ubr1 [10–12]. The enzyme had formerly been found as the ubiquitin ligase of the N-end rule pathway [13]. The cytoplasmic Ssa Hsp70 chaperone machinery and the Hsp40 cochaperone Ydj1 are important for keeping misfolded cytoplasmic substrates soluble and are involved in resolubilization of already precipitated substrate [6].

The *Saccharomyces cerevisiae* heat shock proteins Hsp31, Hsp32, Hsp33 and Hsp34 belong to the DJ-1/ThiJ/PfpI superfamily which includes the human protein DJ-1 (PARK7) as the most prominent member [14–16]. Mutations in the DJ-1 gene are directly linked to autosomal recessive, early-onset Parkinson’s disease. DJ-1 acts as an oxidative stress-induced chaperone preventing aggregation and fibrillation of α-synuclein, a critical factor in the development of the disease [17–20]. *Escherichia coli* (*E*. *coli*) possesses 4 proteins of this superfamily called YhbO, SCRP-27a (ElbB), YajL (ThiJ) and Hsp31 [21, 22]. *E*. *coli* Hsp31, encoded by the *hch*A gene, is a heat-inducible holding chaperone which also possesses weak aminopeptidase activity [23–25]. At higher temperatures Hsp31 exposes hydrophobic domains which serve as binding sites for partially folded client proteins. As holding chaperone Hsp31 complements the DnaK-DnaJ-GrpE system which uses ATP-driven conformational changes to support substrate refolding in *E*. *coli* [25, 26]. In case the client proteins are too severely damaged to be refolded they will be degraded by the heat shock proteases Lon and the ClpXP complex [27]. Interestingly, additional studies could detect an interaction between Hsp31 and ClpA implying an involvement of Hsp31 in the intracellular protein/peptide degradation process mediated by the ClpAP protease [23]. The *E*. *coli* Hsp31 contains a cysteine protease-like catalytic triad consisting of Cys-185, His-186 and Glu-77. The C185A mutation or classical inhibitors of cysteine proteases like iodoacetamide abolish aminopeptidase activity of Hsp31 [23]. A former study revealed the involvement of the catalytic triad in catalysing the detoxification process of methylglyoxal (MG) to lactate [28]. MG is a reactive α-oxoaldehyde which arises as physiological metabolite and may react as toxic electrophile with proteins and nucleic acids [29].

**Fig 1:**
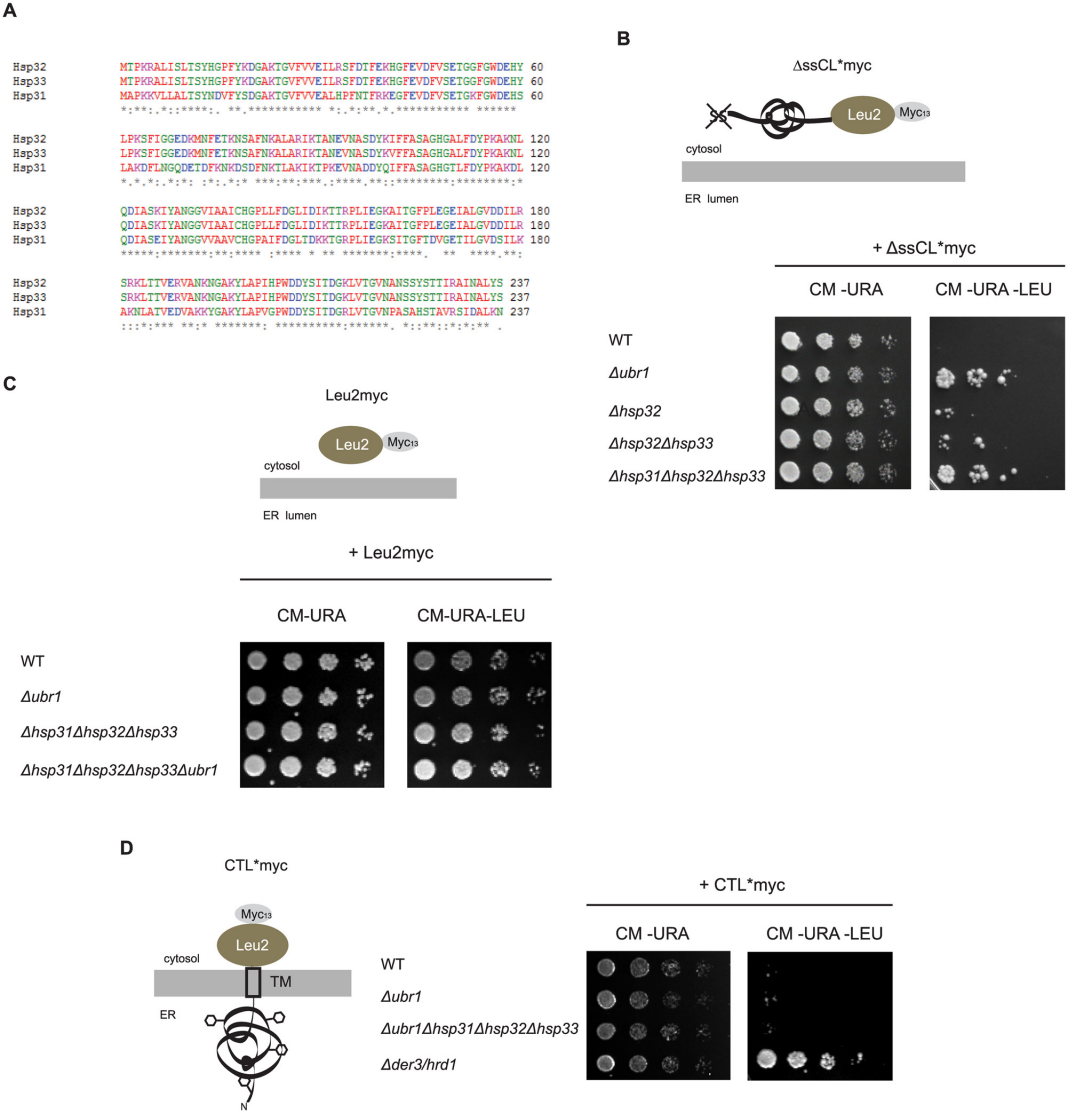
The Hsp31 chaperone family is involved in quality control of misfolded cytoplasmic ΔssCL*myc. (A) Sequence alignment of the three members of the Hsp31 chaperone family in the yeast W303 strain shows an extraordinary sequence homology of 99% between Hsp32 and Hsp33. When compared with Hsp31 they show a sequence homology of about 70%. (B) Growth tests of deletion strains of the E3-Ligase Ubr1, different chaperones of the Hsp31 family and the corresponding wild type (WT) strain, respectively. All strains defective in the *URA3* and *LEU2* genes, harbour a centromeric (CEN) plasmid with the *URA3* marker and expressing ΔssCL*myc under the control of the *PRC1 (CPY)* promoter. ΔssCL*myc is composed of the vacuolar protein carboxypeptidase yscY (CPY) harbouring the point mutation G255R [54]. The protein is additionally deleted in the signal sequence required for transport into the endoplasmic reticulum [6]. In order to perform growth tests it is fused to the enzyme β-isopropylmalate dehydrogenase (Leu2) necessary for leucine biosynthesis. For immunodetection it is C-terminally fused to a c-Myc tag. Cells were spotted in a five fold dilution series on solid selection medium lacking uracil and leucine and, as a control, solely uracil, respectively. (C) Growth tests of cells performed as described in the legend to Fig 1B, but expressing Leu2myc (β-isopropylmalate dehydrogenase C-terminally fused to a c-Myc tag) instead of the terminally misfolded substrate ΔssCL*myc. As ΔssCL*myc, the Leu2myc protein is also expressed under control of the *PRC1* promoter. (D) Growth tests of cells as described in the legend to Fig 1B, but using yeast strains transformed with a *URA3* marker-containing plasmid expressing the ERAD-L model substrate CTL*myc under control of the *GAL4* promoter. CTL*myc consists of the misfolded ER-lumenal carboxypeptidase yscY (CPY*) moiety, the last transmembrane domain of Pdr5, and the cytoplasmic β-isopropylmalate dehydrogenase C-terminally fused to a c-Myc tag (Leu2myc). In contrast to ΔssCL*myc, the CPY* moiety is localized in the ER lumen and therefore glycosylated (black hexagons). The Δ*der3/hrd1* strain defective in ubiquitination of ERAD-L substrates served as control.

The yeast genes *HSP32*, *HSP33* and *HSP34* are located in subtelomeric regions of the genome. This must have occurred through a duplication event of the evolutionary parental *HSP31* gene into a subtelomeric region followed by recombination events which resulted in the additional copies. Hsp32, Hsp33 and Hsp34 share about 99% sequence homology with each other and about 70% homology with Hsp31 (Fig 1A). Hsp31 is a 25.5 kDa protein consisting of 237 amino acids forming a homodimer in solution and adopts an α/β hydrolase fold [30–32]. Yeast Hsp31, 32, 33 and 34 contain the same catalytic triad as the bacterial orthologue but no protease activity could be detected yet. Functional studies revealed that the expression of Hsp31 is induced by oxidative stress under the control of the transcription factor Yap1 [33]. Yap1-mediated gene expression is required for the oxidative stress response [34]. The Hsp31 protein level is furthermore dependent on the growth phase of cells showing increased levels under limiting growth conditions [33]. Additional studies revealed also a Yap1-independent upregulation of Hsp31 transcription when yeast cells are treated with azetidine-2-carboxylic acid (AZC), a toxic proline analogue which causes protein misfolding through incorporation of AZC instead of proline or when nutrients become limiting [33, 35]. A recent study showed that the Hsp31 chaperone family is required for normal entry into diauxic shift and stationary growth phase of yeast cells. In addition, it had been observed that Hsp31 and Hsp32 are localized to stress granules and processing bodies (P-bodies) which constitute storage compartments for translationally silenced mRNAs, formed during cell stress [36–38].

In this study we show that the Hsp31 chaperone family has an influence on the steady state level of irreversibly misfolded cytoplasmic proteins. It appears that the Hsp31 family does not function in exponential growth phase but in late growth phases under conditions of nutrient starvation. Epistasis analysis revealed that the Hsp31 chaperones act in a pathway overlapping with Ubr1-dependent degradation. Both, overexpression of Ubr1 and increase of Ssa Hsp70 activity by overexpression of Ssa1 abolish the dependency of the quality control of the misfolded cytoplasmic model substrate ΔssCL*myc on the Hsp31 chaperone family. The influence of the Hsp31 chaperone family on the steady state level of misfolded cytoplasmic proteins seems to be independent of their function in the oxidative stress response and the vacuolar degradation pathway. This study links the Hsp31 chaperone family to the cytoplasmic protein quality control pathway of misfolded cytoplasmic proteins.

## Materials and Methods

### Media and growth conditions, yeast strains and plasmids

Media preparation as well as molecular biological and genetic techniques were performed using standard methods [39, 40]. All experiments were performed in the genetic background of *Saccharomyces cerevisiae* strain W303. Gene deletion strains were constructed using disruption cassettes replacing the target genes via homologous recombination [41]. Correct integration was confirmed by colony PCR and Southern blotting. The yeast strains used in this study are listed in Table 1. The *URA3* marker-containing plasmid pFE15 used in this study and expressing ΔssCL*myc was described previously [10]. pIA1, the plasmid expressing ΔssCL*myc from a *HIS3* marker-containing plasmid was generated by restriction digest of pFE15 and pRS313 [42] with SalI and NotI and subsequent ligation of the released substrate fragment from pFE15 into pRS313. Leu2myc is expressed from plasmid pIA13. For construction, the *CPY* promoter flanked by XbaI restriction sites (forward primer 5′-atgatctctagaatcgatttccgtatatgatgatac-3′; reverse primer 5′-atgatctctagacatgcatgcagcgtatg-3′) and the fragment *LEU2myc* plus *CPY* terminator flanked by XbaI and SalI (forward primer 5′-ccgtctagacggtctgcccctaagaagatc-3′; reverse primer 5′-tcgcgtcgacggatcccccgggctg-3′) were amplified in two PCR reactions using plasmid pFE15 as template. Both fragments were ligated into plasmid pRS316 cut with XbaI and SalI. The plasmid pSK007 encodes the model substrate CTL*myc [43]. The plasmid pRBUBR1 expresses C-terminally HA-tagged Ubr1 under control of the *ADH1* promoter [44]. For construction of the corresponding plasmid with a *TRP1* instead of the *LEU2* marker, pRBUBR1 and pRS424 [45] were cut with the restriction enzymes PstI and SacI. The resulting *ADH1UBR1HA* fragment was then ligated into the multiple cloning site (MCS) of pRS424 yielding plasmid pIA6. Plasmid pIA10 expressing the inactive RING domain mutant of Ubr1 (Ubr1HA (C1220S)) was obtained by site-directed mutagenesis. For construction of the plasmid pIA11 encoding Ub-ArgΔssCL*myc, PCR was performed using the plasmid pBARUPR [46] as template and the forward primer (5′-agacatgcatgcatgcagattttcgtcaagac-3′) as well as the reverse primer (5′-agacatgcatgctctaccacctcttagccttag-3′) yielding a Ubiquitin (Ub)-Arg-encoding fragment flanked by SphI recognition sites. pBARUPR expresses DHFR-HA-Ub-Arg-e^κ^-HA-URA3. Both pFE15 and the purified fragment were cut with SphI prior to ligation. For generation of plasmid pIA12 arginine was exchanged by isoleucine using site-directed mutagenesis of plasmid pIA11. Plasmid pUB23-R was used for expression of Ub-Arg-βGAL [47]. The plasmid expressing Hsp31 was generated by amplification of Hsp31 including the promoter and terminator region using genomic DNA as template and the forward primer (5′-gatgctcgagagttcagtttgtcatataattatgttt-3′) and the reverse primer (5′-gatggaattcagctcactaagatgcaaataac-3′). The resulting fragment and the plasmid pRS426 [45] were cut with XhoI and EcoRI and ligated, resulting in plasmid pIA30. The pRS426-based plasmid pAM25 codes for histidine-tagged Ssa1 under control of the *GPD* promoter [48].

**Table 1:** Yeast strains used in this study.

Name	Genotype	Source/ Reference
W303-1A	*MATa ade2-1 ura3-1 his3-11*,*15 leu2-3*,*112 trp1-1 can1-100*	[49]
W303-1Ca	*MATa ade2-1 ura3-1 his3-11*,*15 leu2-3*,*112 trp1-1 can1-100 prc1-1*	[50]
W303-1C	*MATα ade2-1 ura3-1 his3-11*,*15 leu2-3*,*112 trp1-1 can1-100 prc1-1*	[50]
YFE9	W303-1Ca *Δubr1*::*loxP*	Frederik Eisele
YFE29	W303-1Ca *Δubr1*::*loxP Δpep4*::*HIS5* ^+^	Frederik Eisele
YIA4	W303-1Ca *Δhsp32*::*HIS5* ^+^	This study
YIA5	W303-1Ca *Δhsp32*::*HIS5* ^+^ *Δhsp33*::*HIS5* ^+^	This study
YIA6	W303-1Ca *Δhsp31*::*loxP Δhsp32*::*loxP Δhsp33*::*loxP*	This study
YIA7	W303-1Ca *Δhsp31*::*loxP Δhsp32*::*loxP Δhsp33*::*loxP Δubr1*::*loxP*	This study
YIA2	W303-1Ca *Δpep4*::*HIS5* ^+^	This study
YIA8	W303-1Ca *Δhsp31*::*loxP Δhsp32*::*loxP Δhsp33*::*loxP Δpep4*::*HIS5* ^+^	This study
YIA9	W303-1Ca *Δhsp31*::*loxP* Δ*hsp32*::*loxP Δhsp33*::*loxP* Δ*ubr1*::*loxP Δpep4*::*HIS5* ^+^	This study
YIA10	W303-1Ca Δyap1::*HIS5* ^+^	This study
YIA11	W303-1Ca *Δubr1*::*loxP Δyap1*::*HIS5* ^+^	This study
YIA12	W303-1Ca *Δhsp31*::*loxP Δhsp32*::*loxP Δhsp33*::*loxP Δyap1*::*HIS5* ^+^	This study
YIA13	W303-1Ca *Δhsp31*::*loxP Δhsp32*::*loxP Δhsp33*::*loxP Δubr1*::*loxP* Δ*yap1*::*HIS5* ^+^	This study
YFE35	W303-1A *Δprc1*::*loxP* Δ*ssa4*::*loxP Δssa3*::*loxP Δssa2*::*loxP ssa1-45* ^*ts*^	Frederik Eisele
YIA1	W303-1A *Δprc1*::*loxP Δssa4*::*loxP Δssa3*::*loxP Δssa2*::*loxP ssa1-45* ^*ts*^ *Δubr1*::*loxP*	This study
YJB009	W303-1C *Δder3/hrd1*::*HIS3*	[51]

### Antibodies

For immunodetection of ΔssCL*myc mouse monoclonal c-Myc antibody was used (Santa Cruz; clone 9E10). The applied dilution was 1:10,000. Horseradish peroxidase-conjugated goat anti-mouse antibody (Jackson Immuno Research) was used as secondary antibody in a 1:10,000 dilution. For detection of 3-phosphoglycerate kinase (PGK) monoclonal PGK antibody was used in a 1:10,000 dilution (Molecular Probes; clone 22C5).

### Growth tests

In order to monitor the steady state levels of protein substrates fused to a protein complementing the genomic auxotrophic marker genes in different yeast strains, the strains were grown overnight in medium selecting for the substrate-encoding plasmid. The OD_600_ values were measured and each cell suspension was diluted with water to an OD_600_ value of 1.0. 100 μl of the different cell suspensions were pipetted into a sterile 96 well plate. 5-fold serial dilutions with water were prepared and a stamp was used for transferring equal amounts of the different dilutions from the 96 well plate onto selection plates selecting for the auxotrophic marker protein-containing substrate. The plates were incubated at 30°C for 2–5 days.

### Colony filter lift assay

Yeast cells expressing β-galactosidase (β-Gal)-containing proteins were grown two days on corresponding agar plates. The cells were then transferred onto a round Whatman filter pre-soaked with growth medium and further grown overnight. The filter with the attached yeast cells was then submerged into liquid nitrogen for cell permeabilization and afterwards incubated overnight at 30°C with an Z-buffer/X-Gal solution (16.1 g/l Na_2_HPO_4_ x 7 H_2_O, 5.5 g/l NaH_2_PO_4_, 0.75 g/l KCl, 0.25 g/l MgSO_4_ x 7 H_2_O, 1 mg/ml X-Gal (Roth; dissolved in DMF)) until the cells turned blue.

### Pulse-chase analysis

Pulse-chase experiments were performed as described previously [52, 53] Briefly, 10 OD_600_ of cells were harvested, washed 3 times and then resuspended in 1 ml of starvation medium lacking methionine. After 50 min of starvation cells were labelled with 0.2 mCi of [^35^S]-methionine (0.37 MBq/μl; PerkinElmer Life Sciences) for 20 min. Chase medium containing 40 mM non-radioactive methionine was then added to the cells and samples were collected at defined time points. Cell breakage, sample preparation and autoradiography were performed as described previously [52, 53].

### Solubility assay

In order to analyse the solubility of protein substrates in different yeast strains 20 OD_600_ of cells were harvested, washed once with 30 mM sodium azide solution, and resuspended in 1 ml of cold sorbitol buffer (0.7 M sorbitol, 50 mM Tris-HCl, pH 7.5, 1.5 μM pepstatin A (Sigma), protease inhibitor cocktail (Roche Diagnostics)). Cells were lysed using glass beads. The crude lysate was precleared by centrifugation at 500 x g for 5 min at 4°C. 400 μl of the supernatant (total protein fraction (T)) were subjected to TCA precipitation. The precipitated proteins were washed once with acetone prior to drying and solubilization in 60 μl of urea sample buffer (40 mM Tris-HCl, pH 6.8, 8 M urea, 5% SDS, 0.1 mM EDTA, pH 8.0, 1.5% β-mercaptoethanol, 100 μg/ml bromophenol blue) at 95°C for 5 min. In addition, 400 μl of the precleared lysate were centrifuged at 21,500 x g for 15 min. The supernatant fraction (S) was subjected to TCA precipitation as described above. After washing the pellet (P) with sorbitol buffer it was dissolved in 60 μl of urea sample buffer at 95°C for 5 min. All samples were subjected to SDS-PAGE and immunoblotting. Temperature-sensitive yeast strains were grown at 25°C and shifted to 37°C for 60 min prior to harvesting and fractionation as described above.

## Results

### Absence of the Hsp31 chaperones causes an increased steady state level of the cytoplasmic model substrate ΔssCPY*Leu2myc

The irreversibly misfolded cytoplasmic model substrate ΔssCL*myc is targeted for proteasomal degradation in a process which is triggered by the ubiquitin ligase Ubr1 [10]. Furthermore, the elimination of different misfolded CPY*-derived cytoplasmic substrates [54, 55] requires the Hsp70-Hsp40 chaperone family proteins Ssa1 and Ydj1 [6]. Here we searched for additional components which might be involved in the elimination process of the misfolded substrate ΔssCL*myc. As candidate proteins we tested the involvement of the Hsp31 chaperone family members Hsp31, Hsp32, Hsp33 and Hsp34 in the protein quality control of ΔssCL*myc. In contrast to the strain S288C which forms the basis of the *Saccharomyces cerevisiae* database (SGD) the *HSP34* gene could not be found in the yeast strain W303 (not shown). All the three Hsp31 family members in the W303 strain show high sequence homology illustrated in Fig 1A.

For testing a possible participation of the Hsp31 family members in the stability and steady state level of ΔssCL*myc, we used a previously invented strategy [52, 56–58]. Yeast W303 cells mutated in the *LEU2* gene are unable to grow on growth medium lacking leucine which is caused by the endogenous *leu2-3*, *112* allele coding for a non-functional Leu2 protein. Expression of ΔssCL*myc which carries a fusion of misfolded ΔssCPY* and the Leu2 protein (β-isopropylmalate dehydrogenase) (Fig 1B) can overcome the growth defect of *leu2-3*,*112* mutant cells when the fusion protein is stable. In contrast to wild type cells this is possible when cells exhibit a defective degradation of the misfolded ΔssCL*myc protein [10]. As can be seen in Fig 1B deletion of the *HSP31*, *HSP32 and HSP33* genes causes a significant enhancement of growth of cells on CM-URA-LEU selection plates. This growth enhancement is strongest when all three members of the Hsp31 family are absent. As expected, similar growth enhancement is observed when Ubr1, the E3 ligase targeting ΔssCL*myc to ubiquitination and thus degradation [10], is absent. There is no difference in growth between the four strains on CM-URA plates selecting only for the presence of the substrate-encoding plasmid pFE15 (Fig 1B). In order to exclude that the natively folded Leu2 domain of the model substrate ΔssCL*myc is responsible for the growth phenotype seen in Fig 1B the strains were transformed with a plasmid encoding the Leu2myc protein. A growth test analogous to the growth test shown in Fig 1B was performed. As can be seen in Fig 1C growth of the strains expressing the Leu2 protein is similar on both types of plates, (i) plates only selecting for the presence of the plasmid (CM-URA plates) and (ii) plates monitoring the Leu2myc steady state level (CM-URA-LEU plates). In order to find out whether the degradation of substrates of the ERAD (endoplasmic reticulum-associated degradation) pathway is also influenced by the Hsp31 chaperone family a growth test was performed using the model substrate CTL*myc. This substrate consists of an ER-lumenal, terminally misfolded carboxypeptidase yscY (CPY*) moiety, the last transmembrane domain of the plasma membrane ATP-binding cassette (ABC) transporter Pdr5, and the cytoplasmic β-isopropylmalate dehydrogenase (Leu2) [52], (Fig 1D). Fig 1D shows that the absence of the E3 ligase Ubr1 has no influence on the stability of CTL*myc. The additional deletion of the genes encoding the Hsp31 chaperone family does also not lead to differences in growth compared to wild type. Der3/Hrd1 represents the E3 ligase involved in degradation of ERAD substrates containing ER-lumenal misfolded domains (ERAD-L substrates), [51]. Therefore, as expected, deletion of *DER3/HRD1* causes stabilization of CTL*myc visible as growth of the CTL*-expressing *Δder3/hrd1* strain on medium lacking leucine (Fig 1D), [51, 52, 56, 57].

### The Hsp31 chaperone family acts in a pathway overlapping with Ubr1-dependent degradation

**Fig 2:**
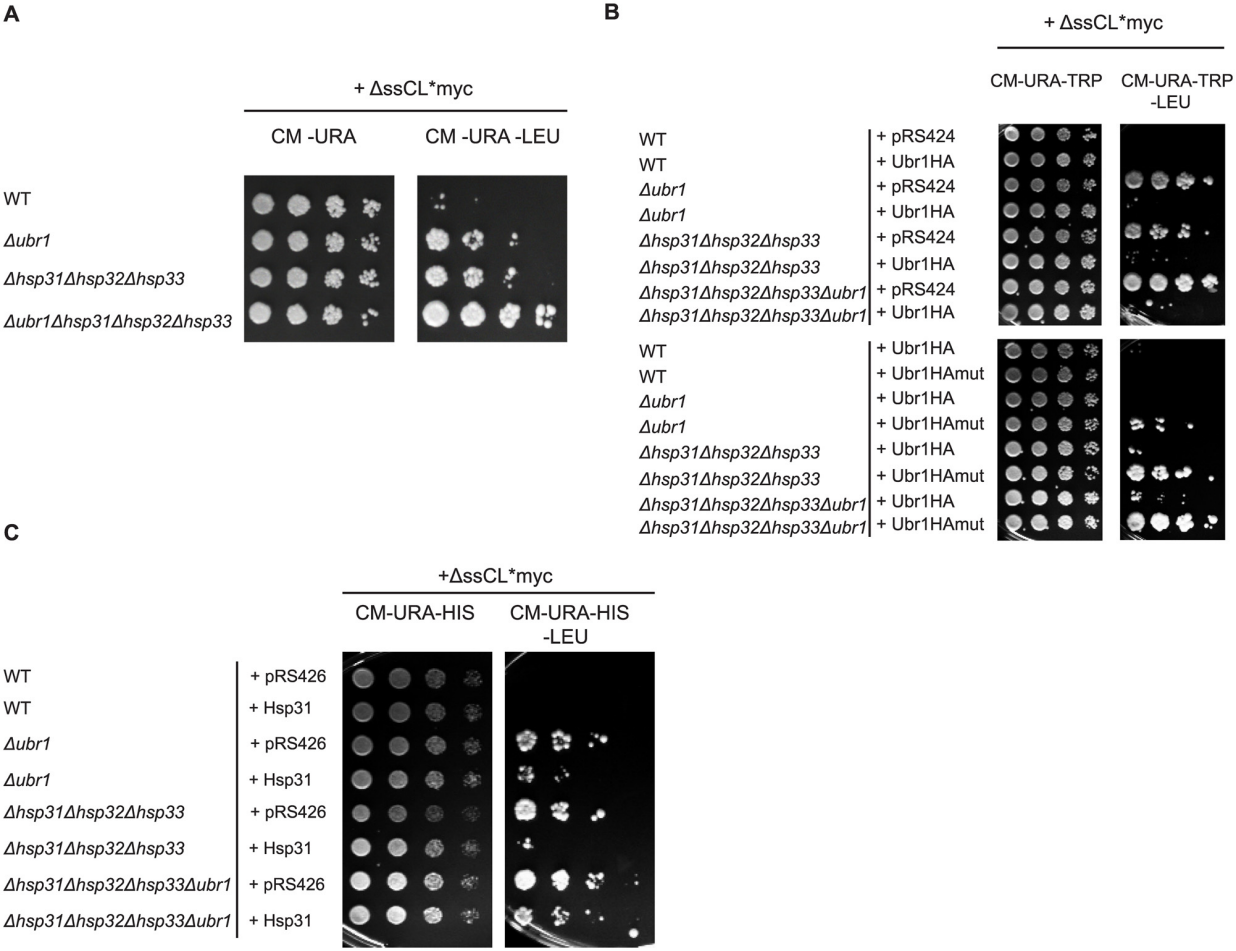
The Hsp31 chaperones function in a pathway overlapping with Ubr1-dependent degradation. (A) Growth tests were performed as described in the legend to Fig 1B using ΔssCL*myc encoded by the plasmid pFE15 as model substrate. Growth of the substrate-expressing *Δubr1* and *Δhsp31-33* strains on medium lacking leucine was compared with growth of the quadruple deletion strain lacking *HSP31*, *HSP32*, *HSP33* and *UBR1* on the same medium. (B) Growth tests were performed using the substrate ΔssCL*myc as described before. The used yeast strains are additionally transformed either with a high copy plasmid encoding C-terminally HA-tagged Ubr1 (Ubr1HA) or the RING mutant Ubr1HA C1220S (Ubr1HAmut). The corresponding empty plasmid pRS424 served as control. All the three plasmids contain a *TRP1* marker for plasmid selection. (C) Growth tests were performed with yeast strains expressing the substrate ΔssCL*myc from a *HIS3* marker-containing plasmid (pIA1). In addition, the strains were either transformed with a high-copy plasmid expressing Hsp31 under control of its own promoter (pIA30) or the corresponding *URA3* marker-containing empty plasmid pRS426.

In a yeast genome-wide interaction study a genetic interaction could be detected between Hsp34, a member of the Hsp31 chaperone family, and the ubiquitin ligase Ubr1 [59]. To test whether Ubr1 and the Hsp31 chaperone family show a genetic interaction with respect to protein quality control of misfolded cytoplasmic proteins an epistasis analysis was performed. The growth tests done as described before were performed to correlate cell growth with the steady state level of the substrate ΔssCL*myc. Growth was compared between the *Δubr1* strain and the *Δhsp31Δhsp32Δhsp33 (Δhsp31-33)* triple mutant as well as the strain harbouring deletions of all four genes. Combination of the *HSP31*, *HSP32* and *HSP33* deletions with the *UBR1* deletion in a strain expressing the misfolded ΔssCL*myc substrate led to a strong enhancement of growth when compared to the *UBR1* single deletion strain or the *Δhsp31-33* triple deletion strain (Fig 2A). From the epistasis analysis one may conclude that Ubr1 and the Hsp31 chaperone family act independently from each other.

When overexpressing Ubr1-HA in *UBR1*-deleted cells expressing the substrate ΔssCL*myc, wild type phenotype is restored and cells cannot grow anymore on medium lacking leucine (Fig 2B). This is also the case, when Ubr1-HA is overexpressed in cells lacking the Hsp31 chaperone family and expressing ΔssCL*myc. This indicates that Ubr1, when overexpressed, abolishes the influence of the Hsp31 chaperone family on the substrate steady state level. The rescue of the *Δhsp31-33* triple mutant phenotype depends on the ubiquitination activity of Ubr1. This can be implicated from the elevated growth of the *Δhsp31-33* strain on medium lacking leucine and expressing a RING mutant of Ubr1 (Ubr1 C1220S), (Fig 2B). Vice versa, the influence of overexpression of Hsp31, one member of the Hsp31 chaperone family, in a strain lacking Ubr1 was examined. Indeed, also expression of Hsp31 in the *Δubr1* strain decreases the growth on medium lacking leucine as compared to the *Δubr1* strain. This confirms the assumption that both, the E3 ligase Ubr1 and the Hsp31 chaperones act in different pathways with respect to the steady state level of ΔssCL*myc (Fig 2C). The functionality of plasmid-encoded Hsp31 is confirmed by the observed reduced growth of the *Δhsp31-33* strain expressing Hsp31 as compared to the *Δhsp31-33* strain harbouring the corresponding empty plasmid pRS426 on medium lacking leucine (Fig 2C).

### N-degrons alter the influence of the Hsp31 chaperones on the steady state level of the substrate ΔssCL*myc

**Fig 3:**
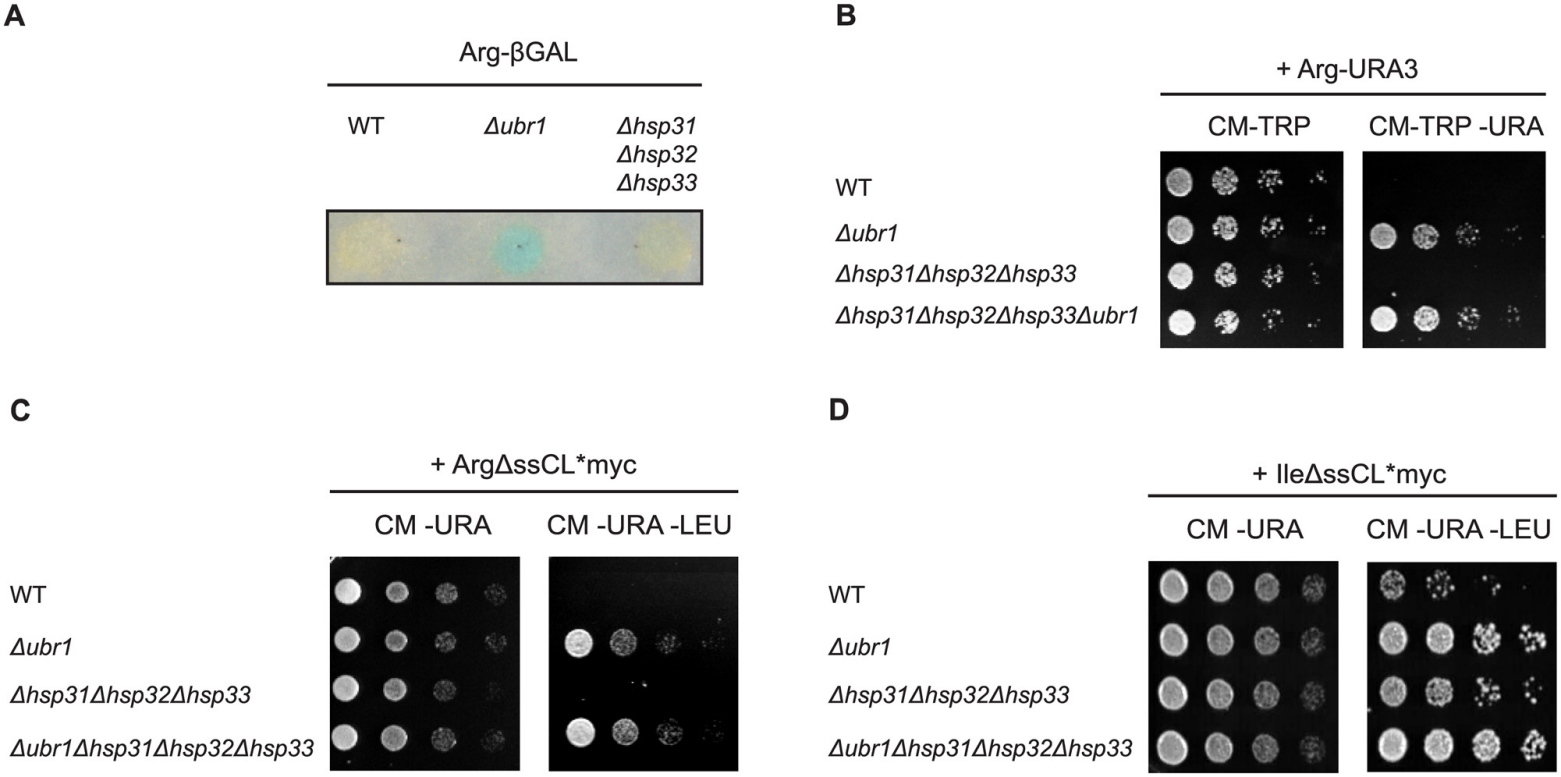
N-degrons alter the influence of the Hsp31 family members on the steady state level of cytoplasmic model substrates in cells. (A) Test of the Arg-βGal levels in cells using a β-galactosidase (βGal) activity assay. The substrate is expressed as a ubiquitin-Arg-βGal fusion protein which is cotranslationally deubiquitinated creating the N-end rule substrate Arg-βGal. βGal activity was measured by incubation of permeabilized cells attached on a filter with X-Gal which is converted into a blue dye by β-galactosidase. (B) Growth tests were performed with strains expressing the type 1 N-end rule substrate Arg-Ura3. Growth of cells on a CM-TRP plate served as control monitoring the presence of the plasmid. (C) Growth tests of cells were performed with strains expressing the misfolded model substrate ΔssCL*myc as in Fig 1B but containing the type 1 N-degron arginine. Initially, the fusion protein ubiquitin-ArgΔssCL*myc is expressed of which ubiquitin is cleaved off. (D) Similar growth tests as shown in Fig 3C were performed with yeast strains expressing ΔssCL*myc containing the type 2 N-degron isoleucine (IleΔssCL*myc), also initially expressed as ubiquitin-IleΔssCL*myc protein.

Ubr1 represents the E3 ligase first discovered as the ubiquitin ligase of the N-end rule pathway which selects substrates for degradation according to the N-terminal amino acid [13, 47, 60]. The substrates used for characterization of the N-end rule pathway were Ub-X-βGal or DHFR-HA-Ub-X-e^κ^-HA-Ura3 which are cotranslationally deubiquitinated and therefore present amino acid X at position one [46, 47]. In case of amino acid X being arginine the resulting substrates Arg-βGal or Arg-e^κ^-HA-Ura3 (Arg-Ura3) enter the N-end rule pathway which leads to proteasomal degradation. The basic amino acid arginine at the N-terminus confers a short half-life to the substrate. In case of Arg-β-Gal the activity of β-galactosidase was tested in a colony filter lift assay as a measure for the steady state level of the substrate (Fig 3A). In case of the substrate Arg-Ura3 growth of cells was tested using the yeast W303 strain carrying the *ura3-1* allele. The resulting absence of uracil biosynthesis in this strain can only be complemented if Arg-Ura3 is not or only slowly degraded (Fig 3B). As can be seen in Fig 3A wild type cells do not show any β-galactosidase activity (yellow colour) indicating rapid degradation of the substrate Arg-βGal. As expected, cells deleted in *UBR1* exhibit β-galactosidase activity, -visible as blue colour-, due to a defective degradation of the substrate. A strain carrying deletions of the genes encoding the Hsp31 chaperone family does not show any β-galactosidase activity indicating that the substrate Arg-β-Gal is as rapidly degraded in these cells as in the wild type strain. In addition, while *UBR1*-deleted cells expressing the substrate Arg-Ura3 grow on medium lacking uracil, the *Δhsp31-33* triple deletion strain behaves like wild type: it is not able to grow (Fig 3B). This shows, that in contrast to the ΔssCl*myc substrate the Hsp31 chaperone family is not involved in elimination of classical rapidly degradable N-end rule substrates.

The terminally misfolded substrate ΔssCl*myc does not represent a classical N-end rule substrate because of the presence of methionine at amino acid position one. According to the Sherman rule methionine is not cut off from proteins carrying bulky amino acids in the second position [61–63]. The second amino acid in ΔssCl*myc is isoleucine (Ile) which, according to the rule, should not be cleaved off by methionine aminopeptidases [61–63]. A recent study showed that methionine followed by a large hydrophobic amino acid could serve as degron for recognition by the E3 ligase Ubr1 [64]. This was also shown to be the case for ΔssCL*myc. When expressing the type 1 N-end rule substrate ArgΔssCL*myc in cells deleted in *UBR1* they grow as well as cells expressing ΔssCL*myc and deleted in *UBR1* (Fig 3C). However, cells expressing ArgΔssCL*myc and carrying deletions of the genes encoding the Hsp31 chaperone family members show wild type behaviour. They do not grow on medium lacking leucine. Obviously, these chaperones are not involved in the elimination of ArgΔssCL*myc, a result which is in contrast to the substrate ΔssCL*myc (Fig 3C). There was also no synthetic phenotype visible when deletions of the genes encoding the Hsp31 family members were introduced into an *UBR1* deletion strain: growth of the *Δubr1* strain expressing ArgΔssCL*myc on medium lacking leucine was comparable to the growth of *Δubr1Δhsp31-33* quadruple mutant cells expressing this substrate. When expressing the substrate ΔssCL*myc exposing the type 2 N-degron isoleucine (IleΔssCL*myc) the steady state level of the corresponding substrate IleΔssCL*myc is increased compared to the steady state level of ArgΔssCL*myc as indicated by growth of the wild type strain expressing IleΔssCL*myc on medium lacking leucine (Fig 3D). The increased steady state level of the type 2 N-degron-containing substrate is consistent with former studies. The substrate Arg-βGal was found to be degraded with a half-life of about 2 min whereas the substrate Ile-βGal shows an about 15 times slower elimination rate [47]. Interestingly, a slight dependence of the steady state level of the substrate IleΔssCL*myc on the Hsp31 chaperone family can be observed in contrast to the type 1 N-degron-containing substrate ArgΔssCL*myc (Fig 3C).

### The Hsp31 chaperones influence the steady state level of ΔssCL*myc in the stationary growth phase of cells

**Fig 4:**
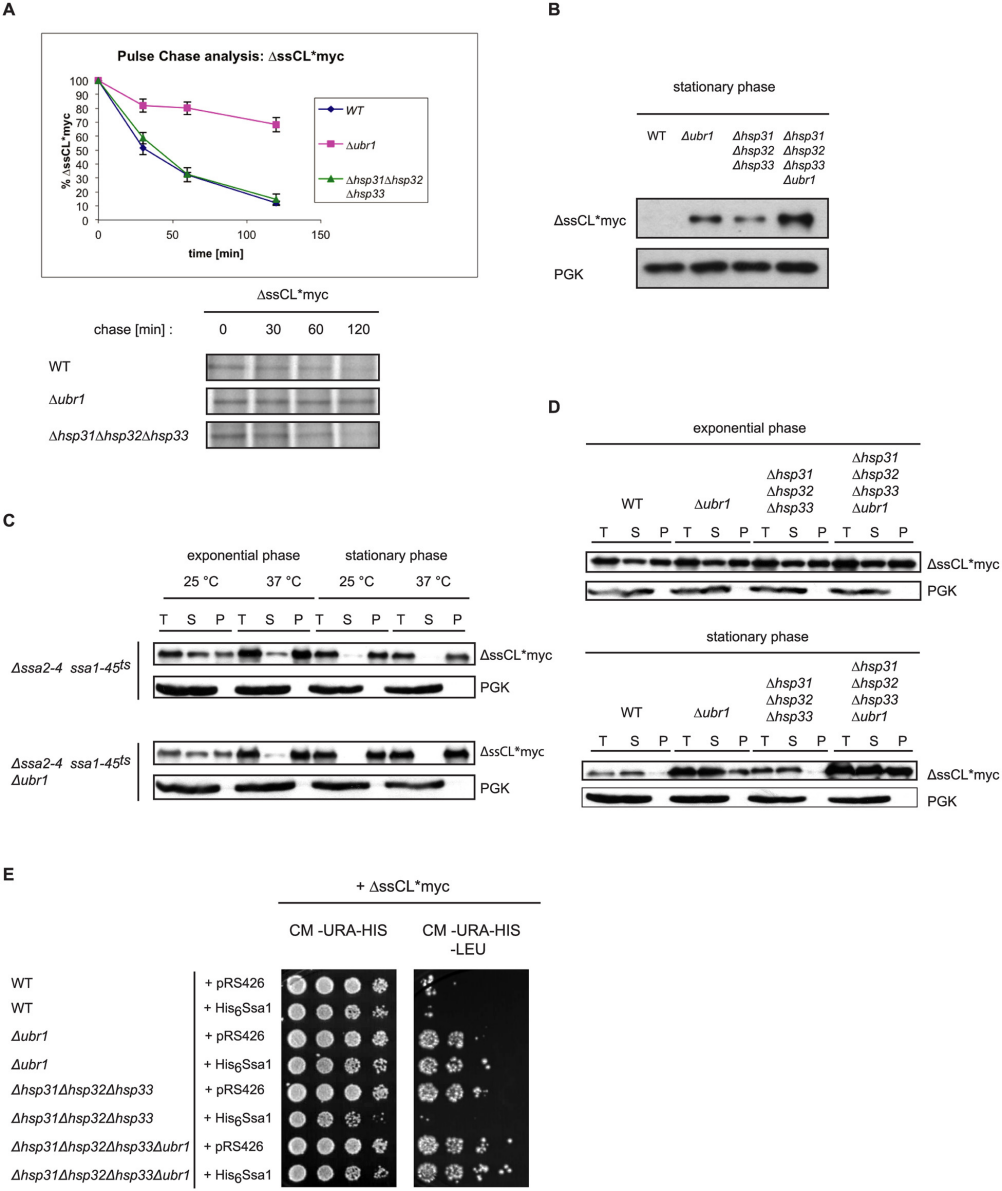
Function of the Hsp31 family in stationary growth phase of cells. (A) Pulse-chase analysis was done with exponentially-growing yeast cells expressing ΔssCL*myc. Cells were lysed at the indicated time points. Proteins were immunoprecipitated, separated by SDS-PAGE and analysed using a Phosphorimager (Storm 860; Molecular Dynamics) and the ImageQuant software (Amersham Biosciences). Plotted data represent the mean values of three independent experiments. Error bars represent the standard error of the mean. (B) Steady state analysis of the amount of the ΔssCL*myc substrate in stationary growth phase. Equal amounts of cells were harvested and cell lysates were subjected to SDS-PAGE followed by immunodetection using c-Myc antibody. As reference protein, 3-phosphoglycerate kinase (PGK) was used. (C) Solubility assays of the misfolded protein ΔssCL*myc expressed in the temperature-sensitive Hsp70 (Ssa) mutant strains *Δssa2Δssa3Δssa4ssa1-45* ^*ts*^ and *Δssa2Δssa3Δssa4ssa1-45* ^*ts*^ *Δubr1*. Cells were grown at 25°C before splitting into two halves. One half of the yeast culture was shifted to 37°C for 1 h prior to harvesting, lysis and fractionation into supernatant (S) and pellet (P) fractions. The total (T) fraction represents the precleared cell lysate. Exponentially growing cells were harvested at an OD_600_ value of 1.0 whereas stationary cells were grown 3 days prior to temperature shift, cell lysis and fractionation. The different fractions were subjected to TCA precipitation prior to SDS-PAGE and immunoblotting using c-Myc antibody for substrate detection. PGK served as loading control and reference for a soluble protein. (D) Solubility assays were performed as described for Fig 4C. Strains defective in either Ubr1 or/and the Hsp31 chaperones were used in this assay. The cells were grown at 30°C and harvested either in exponential phase or stationary phase (72h growth), lysed and subjected to fractionation into supernatant (S) and pellet (P) fractions. The samples were subjected to TCA precipitation, SDS-PAGE and immunoblotting using c-Myc antibody. PGK served as loading control and reference for a soluble protein. (E) Growth tests were performed as described earlier. The used strains express the substrate ΔssCL*myc from a *HIS3*-marker-containing plasmid (pIA1). In addition, the strains were transformed either with the empty plasmid pRS426 containing a *URA3* marker or a pRS426-based plasmid expressing functional histidine-tagged Ssa1 under control of the *GPD* promoter (pAM25).

Previous studies had shown that the Hsp31 chaperones are expressed at diauxic shift upon nutrient limitation whereas in exponential growth phase only a minor amount of Hsp31 chaperones could be detected [33, 36]. We tested the degradation of newly synthesized ΔssCL*myc via pulse-chase analysis during exponential growth phase. As can be seen in Fig 4A the substrate is strongly stabilized in *Δubr1* mutant cells, shown also previously [10]. In contrast, under these exponential growth conditions degradation of ΔssCL*myc proceeds in the *Δhsp31-33* triple mutant strain as in wild type. This indicates that the Hsp31 chaperone family has obviously no function in the degradation of newly synthesized misfolded proteins. The performed growth tests, indicating an impact of the Hsp31 family members on the stability of ΔssCL*myc (Figs 1B and 2A), had been done for several days of incubation until cells enter stationary phase. The diauxic growth phase in which the Hsp31 chaperones start to appear and which precedes stationary phase still allows continuation of cell growth. We therefore determined the steady state levels of ΔssCL*myc in stationary phase by Western blot. Under these conditions hardly any signal of the substrate ΔssCL*myc can be detected in wild type stationary cells. In contrast, in the *Δubr1*, *Δhsp31-33* and the quadruple deletion strain *Δhsp31-33Δubr1* strong substrate signals are visible (Fig 4B). Highest levels of the substrate are detected when deletions of the *UBR1* gene and the genes encoding the Hsp31 chaperone family are combined. The substrate steady state levels in stationary phase as detected via Western blot fit quite well the outcome of the growth test seen in Fig 2A: Highest substrate levels seen in the mutant strain defective in the Hsp31 family and Ubr1 (Fig 4B) coincide with strongest growth of this quadruple mutant on medium lacking leucine (Fig 2A).

In order to examine a possible function of the Hsp31 chaperone family in keeping the substrate ΔssCL*myc soluble we performed solubility assays in exponential and stationary phase of cells. For comparison, we tested the influence of the Hsp70 chaperones of the Ssa type on substrate solubility which are known to keep the similar model substrate ΔssCG* (ΔssCPY*GFP) in a soluble state [6]. When examining the solubility of the substrate ΔssCL*myc in the *Δssa2Δssa3Δssa4ssa1-45* ^*ts*^ strain deficient in three of the four *SSA* genes and carrying a temperature-sensitive allele of *SSA1* (*ssa1-45* ^*ts*^), the results show that after shift of exponentially growing *ssa1-45* ^*ts*^ cells to restrictive temperature the main portion of the substrate is found in the pellet (P) and not in the supernatant (S) fraction (Fig 4C). This indicates that Ssa1 functions in keeping ΔssCL*myc in a soluble state. In stationary phase cells already at permissive temperature the main portion of the substrate is found in the pellet fraction indicating an additional requirement of some other chaperones. After shift of stationary cells to 37°C almost no substrate is detectable anymore in the supernatant (S) fraction. In exponentially growing *ssa1-45* ^*ts*^ cells additionally deleted in *UBR1* even less substrate is detected in the supernatant fraction after temperature shift to 37°C (Fig 4C).

When examining the influence of the Hsp31 chaperones on solubility of ΔssCL*myc in exponentially growing cells no differences in the distribution of the substrate between the pellet and supernatant fractions in wild type and the strain devoid of the Hsp31 chaperones are visible (Fig 4D). The strains lacking the E3 ligase Ubr1 and the quadruple deletion strain devoid of the Hsp31 family and in addition Ubr1 show also similar substrate solubility. In all the four strains tested similar amounts of substrate are detectable in the pellet and supernatant fractions. In stationary phase, the main portion of the ΔssCL*myc substrate expressed in the wild type strain is found in the supernatant fraction (Fig 4D). In contrast to the strains used in the solubility assay shown in Fig 4C, the used strains tested in this assay all harbour the four functional *SSA* chaperone genes *SSA1*, *SSA2*, *SSA3* and *SSA4*. The steady state levels of ΔssCL*myc also differ in the different mutant strains, being highest in the quadruple mutant strain lacking the Hsp31 family and Ubr1. This matches the data of Fig 4B. The amount of ΔssCL*myc protein in the supernatant fraction is not shifted to the pellet fraction when the Hsp31 chaperone family is absent (Fig 4D). This indicates that the Hsp31 family itself is not important for keeping misfolded ΔssCL*myc soluble in stationary phase. When combining the deletion of *UBR1* with the deletions of genes encoding the Hsp31 chaperones the ratio of the amounts of ΔssCL*myc in the supernatant and pellet fractions is shifted towards the pellet fraction (Fig 4D). This indicates some direct or indirect influence of the Hsp31 chaperones on the soluble amount of ΔssCL*myc when Ubr1-triggered degradation is absent. *SSA1* mRNA level is high in exponential growth phase and decreases to nearly undetectable levels at its end. In contrast, *SSA3* mRNA is absent in exponential growth phase and emerges at high levels under nutrient limitation [65, 66]. However, Miller-Fleming and colleagues observed that in cells, deficient in Hsp31 chaperones, *SSA3* mRNA is lowered to a level of about 10% of wild type [36]. Thus, if at least part of the higher ΔssCL*myc protein level of the mutant strain lacking the Hsp31 family were due to a diminished level of Ssa chaperones, a permanent expression of Ssa Hsp70 chaperone activity in this strain should reset growth of the Hsp31 family mutant to wild type behaviour on leucine-lacking medium. This is indeed the case (Fig 4E).

### The influence of the Hsp31 chaperones on steady state level of misfolded cytoplasmic ΔssCL*myc does not depend on vacuolar function or oxidative stress response

**Fig 5:**
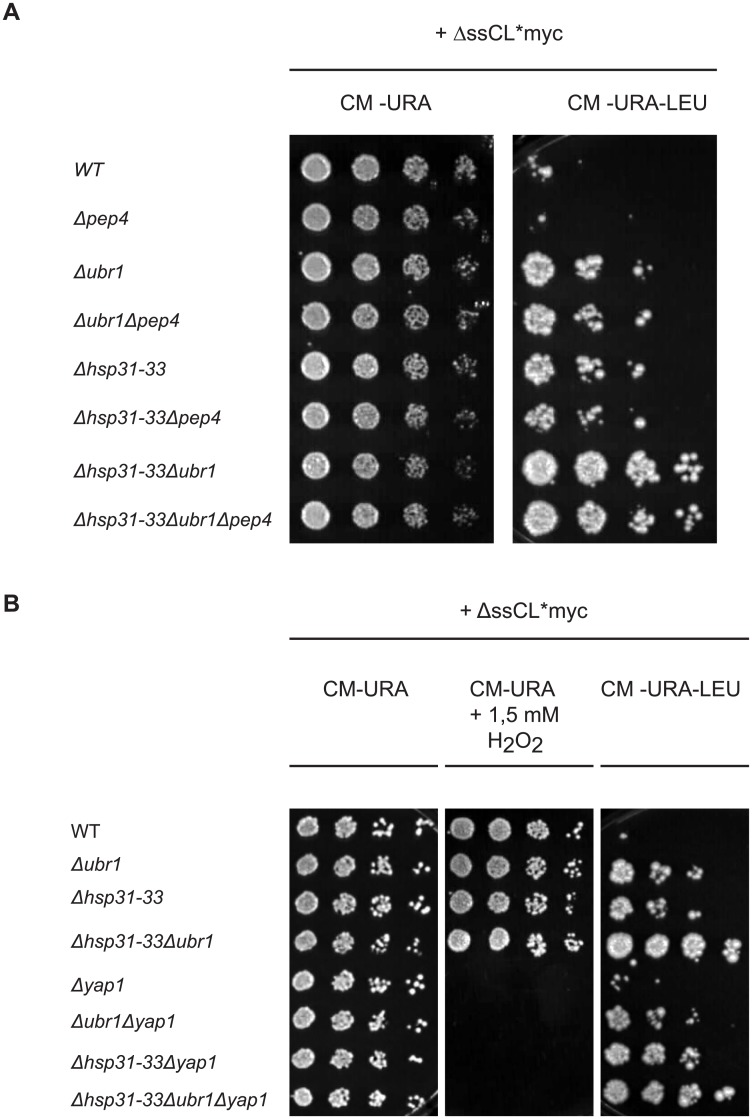
Diminishing vacuolar function or the oxidative stress response does not alter the dependency of the steady state level of ΔssCL*myc on the Hsp31 family. (A) Growth tests were performed as described above using yeast strains carrying a *PEP4* deletion. All strains were transformed with the plasmid pFE15 encoding the model substrate ΔssCL*myc. Medium lacking uracil served as control selecting only for the presence of the plasmid. (B) Yeast strains transformed with the plasmid pFE15 encoding ΔssCL*myc were used for the growth tests performed as described above. Yeast strains possessing the *YAP1* gene were compared with *YAP1* deletion strains. Defective growth of cells on plates containing 1.5 mM hydrogen peroxide served as verification for the absence of Yap1. Medium lacking uracil served as control for selection of cells carrying the plasmid pFE15.

In order to test the influence of the vacuole in the protein quality control pathway concerning the model substrate ΔssCL*myc, strains were constructed which lack the vacuolar aspartyl protease yscA, also called Pep4. The enzyme is responsible for maturation of vacuolar proteinases as well as for general protein degradation in the vacuole [67, 68]. Growth tests were performed comparing yeast strains harbouring the *PEP4* gene with strains deleted in *PEP4*. All strains express the cytoplasmic model substrate ΔssCL*myc. As can be seen in Fig 5A the deletion of *PEP4* does not increase growth of the wild type strain expressing the substrate. In addition, combining the *PEP4* deletion with deletion of the genes encoding the Hsp31 chaperones or/and the *UBR1* gene the growth phenotypes on medium lacking leucine are unchanged compared to the strains harbouring *PEP4* (Fig 5A). Therefore, the vacuole does not play a role in the quality control of ΔssCL*myc.

Upon nutrient starvation the cell produces more and more energy from mitochondrial respiration which also increases the production of ROS responsible for oxidative stress. In a former study it had been shown that Hsp31 is expressed upon oxidative stress in a Yap1-dependent manner [33]. *YAP1* codes for a transcription factor essential for oxidative stress tolerance [34, 69]. Therefore it was interesting to test if the growth effects seen for the *HSP31-33* deletion strain transformed with a ΔssCL*myc-encoding plasmid were due to a defective oxidative stress response. Growth tests were performed with strains lacking genes encoding the E3 ligase Ubr1 or the Hsp31 chaperone family and additionally deleted in *YAP1*. Here, the plate containing hydrogen peroxide does not allow any growth of strains containing the *YAP1* deletion, confirming the identity of the yeast strains. Growth of the different yeast strains on medium lacking leucine is similar, independent of the deletion of *YAP1*. Only a slight enhancement of growth of the strain lacking the Hsp31 chaperone members compared to the *Δubr1* strain can be observed on plates lacking leucine when Yap1 is missing (Fig 5B). In summary, the results indicate a function of the Hsp31 chaperone family in quality control of ΔssCL*myc independent of the oxidative stress response.

## Discussion

Quality control of misfolded proteins in the cytoplasm is exerted by an array of chaperones and cochaperones, components of the ubiquitin system and the proteasome. The major ubiquitin ligase triggering polyubiquitination of misfolded cytoplasmic proteins in yeast cells is Ubr1 [10–12]. The Hsp70 chaperones of the Ssa type are also central in the degradation pathway [6, 70]. The Hsp70 Ssa1 had turned out to be an essential chaperone in the elimination process. One function in protein quality control resides in preventing aggregation of misfolded proteins for subsequent degradation [6, 71]. It is furthermore able to solubilize aggregated proteins [6]. The finding that the chaperones of the Hsp31 class in yeast are orthologues of the human DJ-1 (PARK7) gene, mutations of which are linked to autosomal recessive early-onset parkinsonism [17, 18, 72] led us search for a function of these chaperones in the quality control pathway of misfolded cytoplasmic proteins. In the yeast strain background S288C the genes of the four Hsp31 family members,—*HSP31*, *HSP32*, *HSP33* and *HSP34* -, are present. In the W303 background used by us we could find only three *HSP* genes of this type. *HSP34* is missing. The Hsp31 chaperone family is expressed under oxidative stress, upon diauxic shift and under limiting nutrient conditions [33, 36]. For studying the involvement of the Hsp31 chaperone family in protein quality control of misfolded cytoplasmic proteins we used a formerly established growth test [52, 57, 58]. It rests on the misfolded cytosolic fusion protein (ΔssCPY*Leu2myc) carrying the Leu2 protein (β-isopropylmalate dehydrogenase) expressed in a strain deficient in the *LEU2* gene. Such a strain is only able to grow, when the misfolded protein exhibits an increased steady state level. The validity of this growth test as a qualitative measure for the presence of the misfolded protein is shown by a correlation of cell growth with an immunological detection of the protein substrate content under limiting nutrient conditions (Figs 4B and 2A). Wild type cells do not contain considerable ΔssCL*myc substrate in stationary phase and can hardly grow on plates lacking leucine, while strains defective in *HSP31* family genes contain increased substrate amount and do grow. This indicates a clear involvement of the chaperones in the regulation of the amount of the substrate protein. The Hsp31 family members work in an additive manner. The more of the chaperone family members are missing, the better is cell growth on medium lacking leucine (Fig 1B). Obviously, Hsp31, Hsp32 and Hsp33 exert similar functions in the cell concerning quality control of ΔssCL*myc. The ubiquitin ligase Ubr1 had been shown to target ΔssCL*myc to degradation via the proteasome [10]. A quadruple mutant defective in Ubr1 and in the three Hsp31 family members shows an additive effect concerning growth, (Fig 2A), and substrate amount under nutrient limitation (Fig 4B). From this result one may conclude that Ubr1-triggered substrate degradation and substrate disappearance on the basis of the action of the Hsp31 family seems to be two different events. However, as overexpression of Ubr1 in a mutant strain deleted in the Hsp31 family or, vice versa, overexpression of Hsp31 in a *UBR1*-deleted mutant drastically reduces growth (Figs 2B and 2C), the two paths seem to be able to interchange. When more Ubr1 ligase is available the substrate can be cleared despite the absence of the Hsp31 chaperone family. Ubr1 had been shown to be the ubiquitin ligase of the N-end rule pathway [13, 47, 60]. Indeed, when the strong type 1 N-degron arginine was N-terminally fused to the terminally misfolded substrate it becomes completely dependent on the Ubr1 ligase without any further influence of the Hsp31 chaperone family (Fig 3C). This is most likely caused by binding of the substrate to Ubr1 with high affinity, by this circumventing the Hsp31 linked process. Fusion of the type 2 N-degron isoleucine to the N-terminus of ΔssCL*myc causes an increase of the steady state level even in the wild type strain (Fig 3D). This observation is consistent with the half-lives of β-galactosidase (βGal) based N-end rule substrates. Whereas Arg-βgal shows a half-life of 2 min the substrate Ile-βGal is degraded with a half-life of 30 min [47]. The N-degron isoleucine may therefore also increase the retention time of Ile-ΔssCL*myc. This increased retention time and therefore the increased steady state level of Ile-ΔssCL*myc may be responsible for the slight growth dependency of cells on the Hsp31 family (Fig 3D). The influence of the Hsp31 chaperones on the steady state level of the substrate ΔssCL*myc which does not contain isoleucine as a classical N-degron but methionine followed by isoleucine as second amino acid, is much stronger. This again indicates that methionine is not cut off from the N-terminus as postulated by the Sherman rule. In summary, substrates containing strong N-degrons like arginine overcome the dependency on the Hsp31 family dependent quality control pathway independent of their folding status.

When following the degradation of the terminally misfolded ΔssCL*myc protein via pulse- chase analysis in logarithmic growth phase of cells, *UBR1* deletion mutants exhibit a strong cessation of the substrate degradation rate. Mutants of the Hsp31 family show the same fast degradation kinetics of the substrate as wild type (Fig 4A). This is not surprising as the Hsp31 chaperones are hardly expressed in exponentially growing cells as shown for the Hsp31 member [33]. As a considerable labelling of ΔssCL*myc is not possible in stationary phase cells we measured the steady state level of the substrate in wild type and mutant cells. Here a function of the Hsp31 chaperone family on the cellular amount of ΔssCL*myc becomes visible (Fig 4B).

It is known that the vacuolar degradation pathway becomes exceedingly important in stationary phase. However the growth test as a measure for the ΔssCL*myc substrate content did not show any differences when comparing cells deleted in *PEP4*, -the gene encoding the vacuolar proteinase activating proteinase yscA-, with the respective reference strains (Fig 5A). This indicates that vacuolar proteolysis is not involved in the quality control of the substrate.

Mutants of the Hsp31 family impair autophagy induction under carbon starvation [36]. However, since vacuolar proteolysis, the terminal fate of autophagocytosed proteins, does not play a role in degradation of ΔssCL*myc, the enhanced steady state level of the substrate in mutant cells of the Hsp31 family cannot be due to Hsp31 family function in autophagy.

Hsp31 is induced under conditions of oxidative stress which is under control of *YAP1* encoding a transcription factor essential for oxidative stress tolerance [34]. Deletion of *YAP1* does not lead to gross alterations in growth of Hsp31 mutant cells expressing the misfolded ΔssCL*myc substrate (Fig 5B). This indicates that the function of the Hsp31 chaperone family in quality control of ΔssCL*myc is independent of the oxidative stress response.

Yeast expresses four Ssa chaperones (Ssa1, Ssa2, Ssa3, Ssa4) of the Hsp70 type possessing high sequence similarity. The mRNA levels of *SSA1* and *SSA2* increase in logarithmic phase and decrease when cells reach limiting growth conditions. In contrast, *SSA3* mRNA is only detected after diauxic shift of cells and accumulates to high levels when cells enter stationary phase [65, 66]. Interestingly, deletion of each Hsp31 family member leads to a reduction of *SSA3* mRNA level of about 90% [36]. Ssa1 has been shown to be strongly required for keeping misfolded and orphan proteins soluble for subsequent proteasomal degradation [6, 71]. Also a considerable portion of the misfolded substrate ΔssCL*myc used in this study is kept in a soluble form in exponentially growing mutant cells deleted in the genes encoding the Hsp70 chaperones Ssa2, Ssa3 and Ssa4 and carrying the temperature-sensitive *SSA1* allele *ssa1-45* ^ts^. At the restrictive temperature of 37°C most of the substrate material is found in the pellet in exponentially growing mutant cells due to the temperature-induced inactivity of Ssa1 (Fig 4C). In contrast, in stationary phase mutant cells already at the permissive temperature of 25°C most ΔssCL*myc is found in the pellet fraction. Temperature-induced inactivation of Ssa1 leads to nearly the same result (Fig 4C). This can be explained by the fact that in exponentially-growing cells at permissive temperature Ssa1 is expressed and is able to keep the substrate soluble while in stationary phase at permissive temperature expression of a functional Ssa1 has ceased while Ssa3 which usually appears in stationary phase is deleted in this strain [65]. This observation fits to the solubility assay performed with a stationary wild type strain expressing all four members of the Ssa chaperones. Here, most of the expressed substrate ΔssCL*myc is found in the supernatant fraction, even more than in exponential phase (Fig 4D). This indicates enhanced expression of Ssa members other than Ssa1, most likely Ssa3. The Hsp31 family members do not seem to have a function by themselves in keeping substrate soluble as deletion of these chaperones in a strain wild type for all Ssa chaperones does not lead to any accumulation of insoluble (P) misfolded ΔssCL*myc in stationary phase cells. (Fig 4D). Only when *UBR1* is deleted in addition, considerable precipitate is formed indicating accumulation of an overwhelming amount of substrate which cannot be ubiquitinated and thus cannot be degraded. This high amount of substrate can obviously not be kept soluble anymore by the small amount of Ssa3 [36] which seems to be expressed in stationary phase in mutants deleted in the genes encoding the Hsp31 family. Indeed, when the Hsp70 member Ssa1 is constantly expressed, growth of a mutant lacking the Hsp31 chaperone family is reverted to wild type level. When Ubr1 is missing at the same time as the Hsp31 family the substrate material cannot be degraded and the strain grows (Fig 4E). It has been shown that Ssa Hsp70 activity is crucial for elimination of a similarly misfolded protein as ΔssCL*myc, ΔssCG* [6], a process which is partly dependent on Ubr1 function [11]. Also, Ssa Hsp70 activity is required for Ubr1-dependent degradation of an orphan protein, Fas2 [71]. From this one may assume that Ssa Hsp70 activity is generally important for Ubr1-dependent degradation of misfolded proteins. Thus, downregulation of Ssa Hsp70 activity upon deletion of the Hsp31 chaperone genes must disturb this process considerably. On the other hand, reduced growth of cells deficient in Ubr1 and overexpressing Hsp31 (Fig 2C) indicates additional Hsp31 function in protein quality control independent of the Ubr1 pathway as previously shown (Fig 2A).

DJ-1, the human orthologue of the Hsp31 chaperone family when mutated, causes α- synuclein aggregation followed by apoptosis of dopaminergic neurons in the brain [17–20]. With respect to α-synuclein, mutations of DJ-1 may cause α-synuclein aggregation due to a similar downregulation of important chaperones which keep α-synuclein in a soluble form when functional. Such a mechanism might be an additional factor for triggering Parkinson’s disease.
